# Privacy Preserving Probabilistic Record Linkage (P3RL): a novel method for linking existing health-related data and maintaining participant confidentiality

**DOI:** 10.1186/s12874-015-0038-6

**Published:** 2015-05-30

**Authors:** Kurt Schmidlin, Kerri M. Clough-Gorr, Adrian Spoerri, for the SNC study group

**Affiliations:** Institute of Social and Preventive Medicine (ISPM), University of Bern, Finkenhubelweg 11, CH-3012 Bern, Switzerland; Section of Geriatrics, Boston University Medical Center, 88 East Newton St., Boston, MA 02118 USA

**Keywords:** Patient confidentiality, Privacy, Probabilistic record linkage, Record linkage, Bloom filters

## Abstract

**Background:**

Record linkage of existing individual health care data is an efficient way to answer important epidemiological research questions. Reuse of individual health-related data faces several problems: Either a unique personal identifier, like social security number, is not available or non-unique person identifiable information, like names, are privacy protected and cannot be accessed. A solution to protect privacy in probabilistic record linkages is to encrypt these sensitive information. Unfortunately, encrypted hash codes of two names differ completely if the plain names differ only by a single character. Therefore, standard encryption methods cannot be applied. To overcome these challenges, we developed the Privacy Preserving Probabilistic Record Linkage (P3RL) method.

**Methods:**

In this Privacy Preserving Probabilistic Record Linkage method we apply a three-party protocol, with two sites collecting individual data and an independent trusted linkage center as the third partner. Our method consists of three main steps: pre-processing, encryption and probabilistic record linkage. Data pre-processing and encryption are done at the sites by local personnel. To guarantee similar quality and format of variables and identical encryption procedure at each site, the linkage center generates semi-automated pre-processing and encryption templates. To retrieve information (i.e. data structure) for the creation of templates without ever accessing plain person identifiable information, we introduced a novel method of data masking. Sensitive string variables are encrypted using Bloom filters, which enables calculation of similarity coefficients. For date variables, we developed special encryption procedures to handle the most common date errors. The linkage center performs probabilistic record linkage with encrypted person identifiable information and plain non-sensitive variables.

**Results:**

In this paper we describe step by step how to link existing health-related data using encryption methods to preserve privacy of persons in the study.

**Conclusion:**

Privacy Preserving Probabilistic Record linkage expands record linkage facilities in settings where a unique identifier is unavailable and/or regulations restrict access to the non-unique person identifiable information needed to link existing health-related data sets. Automated pre-processing and encryption fully protect sensitive information ensuring participant confidentiality. This method is suitable not just for epidemiological research but also for any setting with similar challenges.

## Background

Record linkage of existing individual health-related data is a time and cost efficient way to answer important epidemiologic research questions. For example, survival studies linking federally collected mortality data with existing clinical cohorts or population-based disease registries provide quick thorough participant follow-up without cumbersome and expensive patient contact [1, 2]. Given rapidly increasing low cost computing power, these types of digital data (even very large datasets) can be easily combined for new research objectives using record linkage.

Unfortunately, today much of the available existing health-related data is primarily collected for specific purposes under strict privacy protection regulation. A major challenge for data reuse is that regulations commonly prohibit disclosure of discriminating personal identifying information ([PII], e.g. name, address, social security number [SSN], date of birth [DOB]). PII is any data that is considered potentially unique to a person’s identity. Only recently in Switzerland was a SSN introduced nationally. Although older Swiss datasets include non-unique PII and newer datasets have SSN, neither can be used across settings due to legal restrictions. This creates an intractable challenge for researchers who want to take advantage of the efficiencies of reusing existing health-related data [3, 4].

Generally, probabilistic record linkage methodology is applied [5–7] to combine two (or more) datasets if no unique identifier is available. Records are linked based on commonly stored non-unique variables available in both datasets. Commonly used linkage variables are names, DOB, address, sex, nationality and marital status. For each pair of records, the likelihood of referring to the same individual is calculated using weights for each linkage variable, allowing for typical data errors. A solution to protect privacy in probabilistic record linkages is to encrypt PII. Unfortunately, encrypted hash codes of two names differ completely if the plain names differ only by a single character. Therefore, standard encryption methods cannot be applied. Encryption methods specifically for probabilistic record linkage have been widely researched [8–13]. Bloom filters [14–19] have been shown to be a useful and efficient privacy preserving encrypting method suitable for probabilistic record linkage projects. They enable calculation of the similarity of encrypted variables and therefore allow linkage in spite of typos and other errors existing in any dataset.

In response to these challenges we developed Privacy Preserving Probabilistic Record Linkage (P3RL). The aim of P3RL is to reliably link individual health-related data for new research objectives without breach of participant confidentiality (i.e. revealing PII). P3RL has the potential to transform epidemiologic research by making available vast amounts of health-related data anonymously and thus heretofore not previously accessible. In this paper we describe the P3RL method developed for use in health-related research settings in Switzerland. However, this work is applicable in any settings facing similar challenges.

## Methods

### Setting

**Fig. 1:**
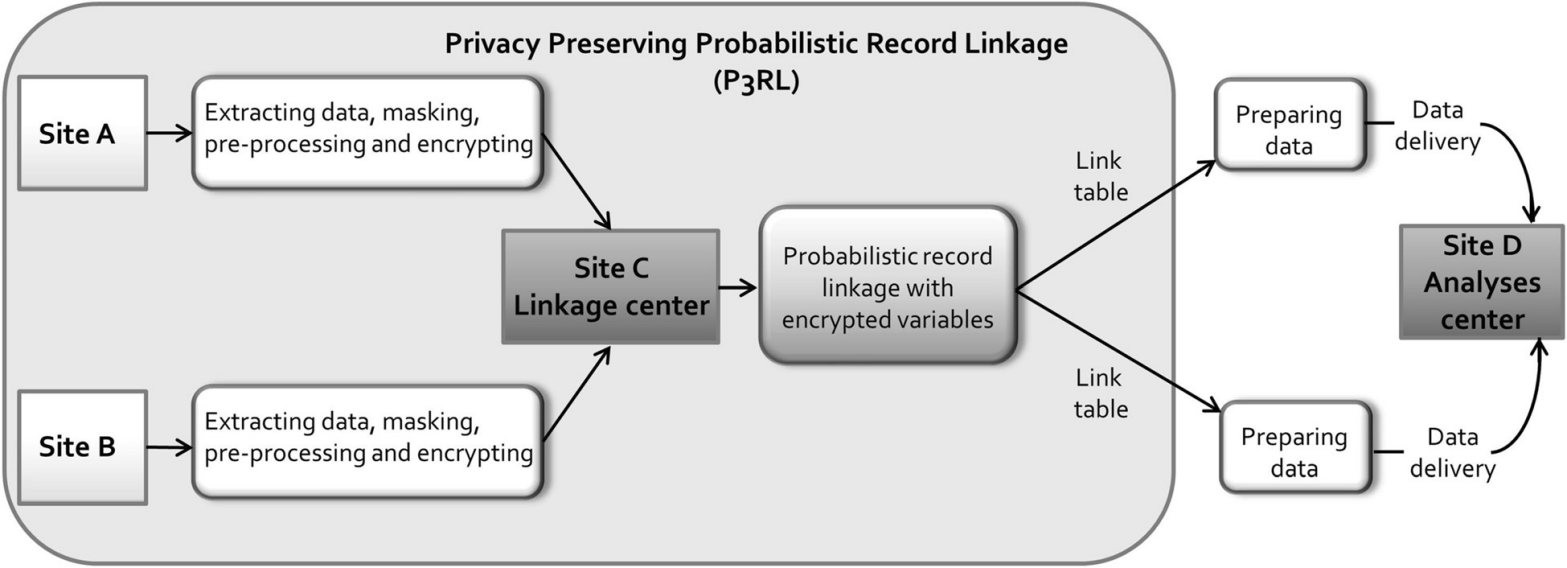
Basic steps of Privacy Preserving Probabilistic Record Linkage (P3RL)

Figure 1 gives an overview of the P3RL method. P3RL is appropriate for settings where sites collect individual health-related data on the same persons without a common unique identifier (ID) and with regulations restricting access to non-unique PII. In this paper, we use a two site example (site A&B). In P3RL, probabilistic record linkage with encrypted non-unique PII (e.g. names, DOB, date of death [DOD], address) and plain linkage variables (e.g. gender, marital status, nationality) are used to combine the data from sites A and B. P3RL utilizes a trusted linkage center (site C) [20]. The linkage center is an independent partner with stringent ethical guidelines and up-to-date privacy safeguards. It performs P3RL using only linkage variables (encrypted PII and plain demographic variables without health-related information). Site C is not involved in data collection or analyses, has no direct access to the individual records at site A or B and never sees PII in plain text. This is in keeping with Kelman et al.’s best practice protocol [21]. The output from P3RL is a link table containing only mapped site IDs (e.g. site A ID 1234 = site B ID 789). The link table is used to combine the records from the individual sites into a dataset tailored for the defined research objectives. The analysis of the linked data – which is not covered by this paper – can either be done at site A, B or at a specific analysis center (site D), depending on the project design and legal permissions.

**Fig. 2:**
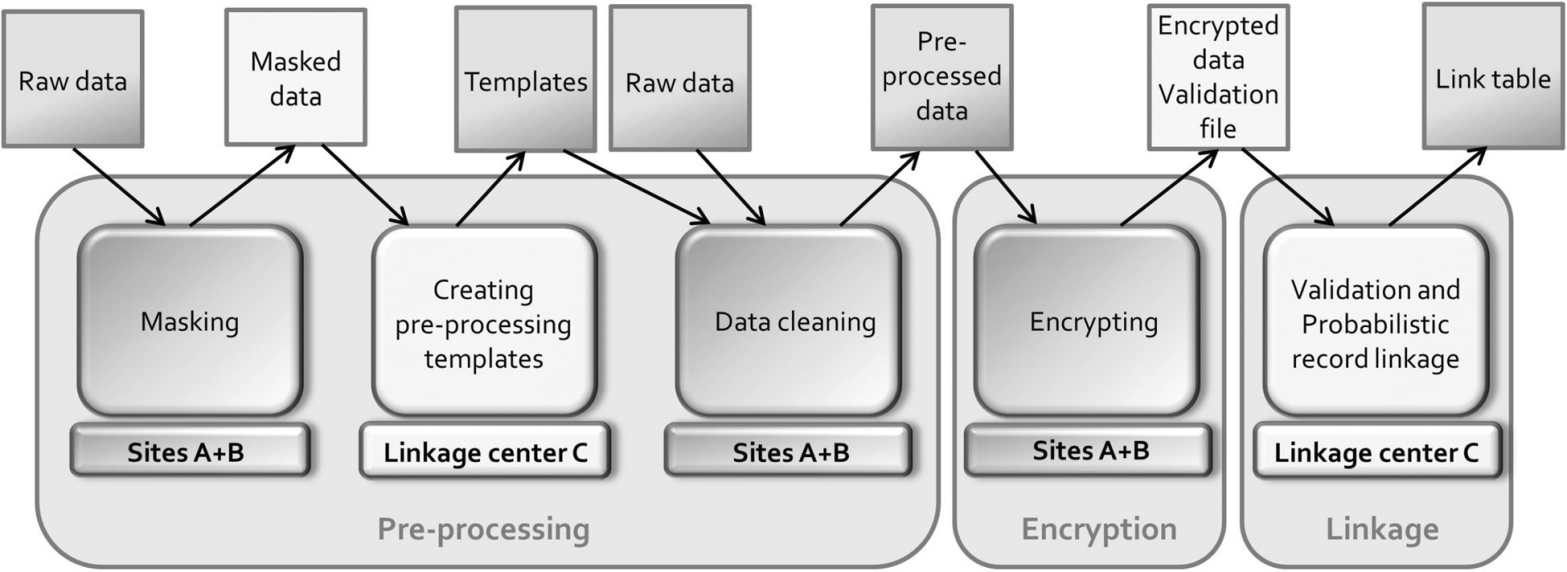
Flowchart of Privacy Preserving Probabilistic Record Linkage (P3RL) methods

Figure 2 shows the flow of data between sites and the sites responsible for the individual steps included in our P3RL method. P3RL consists of three main steps: pre-processing, encryption and probabilistic record linkage. Data pre-processing and encryption are done at the data custodian sites (site A and B) by authorized local personnel. Creating pre-processing and encryption templates, encryption validation and probabilistic record linkage are done at the linkage site (site C).

### Pre-processing

Pre-processing is a crucial step in record linkage [22–30]. The aim of pre-processing is to harmonize the linkage variables at each site to make them directly comparable and thus easier to link. Pre-processing includes three steps: masking (site A&B), creating pre-processing templates (site C) and data cleaning (site A&B). Using masking, the linkage center creates custom pre-processing templates based on the data structure at each site. The templates are supplied to the individual sites allowing them to perform standardized data cleaning procedures that result in linkage variables with similar data quality and harmonized formats.

#### Masking

**Fig. 3:**
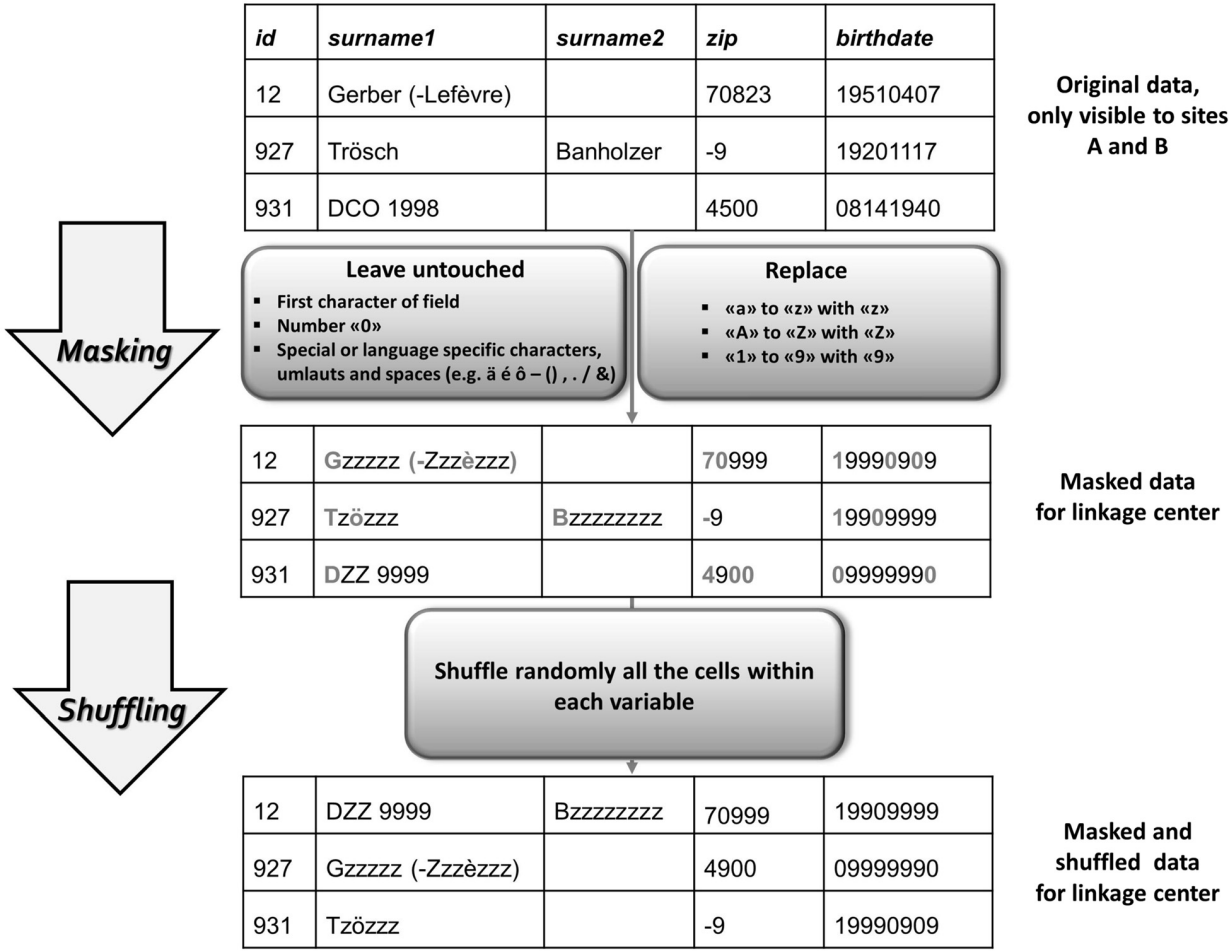
Example of Privacy Preserving Probabilistic Record Linkage (P3RL) masking and shuffling procedures

Masking is used to disclose the individual site data structures to site C without revealing PII. The masked data are used to create the site-specific pre-processing templates. Data for building pre-processing templates are exported to site C as masked alone or masked and additionally shuffled depending on site restrictions. Figure 3 illustrates examples of the masking and shuffling procedures.

The aim of masking is to alter the plain text variables so they are no longer readable. Masking replaces numeric characters between “1 and 9” with “9”, lower case alpha characters “a to z” with “z” and upper case “A to Z” with “Z”. Some characters are left untouched. For example, first characters of fields, numeric character zero, special or language specific characters (e.g., ä é - () &) and spaces are unchanged. Masking of linkage variables is performed at the individual sites based on a pre-determined sample number of records or the entire population (depending on project-specific restrictions). Masking informs the data cleaning procedures by hinting at data errors, like numbers in name fields, characters in a numeric field or special codes for missing data (e.g., 9, 99, −, .) and reveals language of text, number of names (surnames, first names) in a single variable, separators, special characters (e.g. language specific) and date formats.

Shuffling randomly rearranges all cells within each variable to further increase confidentiality. For example, masked variables such as surname or DOB from a single record get randomly assigned to different records (i.e. masked linkage variables in the shuffled record no longer belong to the same person, Fig. 3). If only masking is used, it is possible for site C to give sites feedback about inconsistencies in the raw data so the data can be updated before the next step in P3RL is performed.

Figure 3 demonstrates the masking and shuffling procedures. For example, the masked data reveal that ID 12 has two surnames in the same variable, ID 927 has two surnames in separated fields and uses language specific umlauts. In ID 931 a name with numbers, which might stand for “DCO 1998 = Death certificate only – year 1998”, is disclosed and can be corrected during data cleaning. ID 927 has a 1-digit number prefixed by a dash for the zip code, which could be a code for missing value. The masked birthdates uncover different order of year, month and day, which has to be processed into a single format.

#### Pre-processing templates

**Fig. 4:**
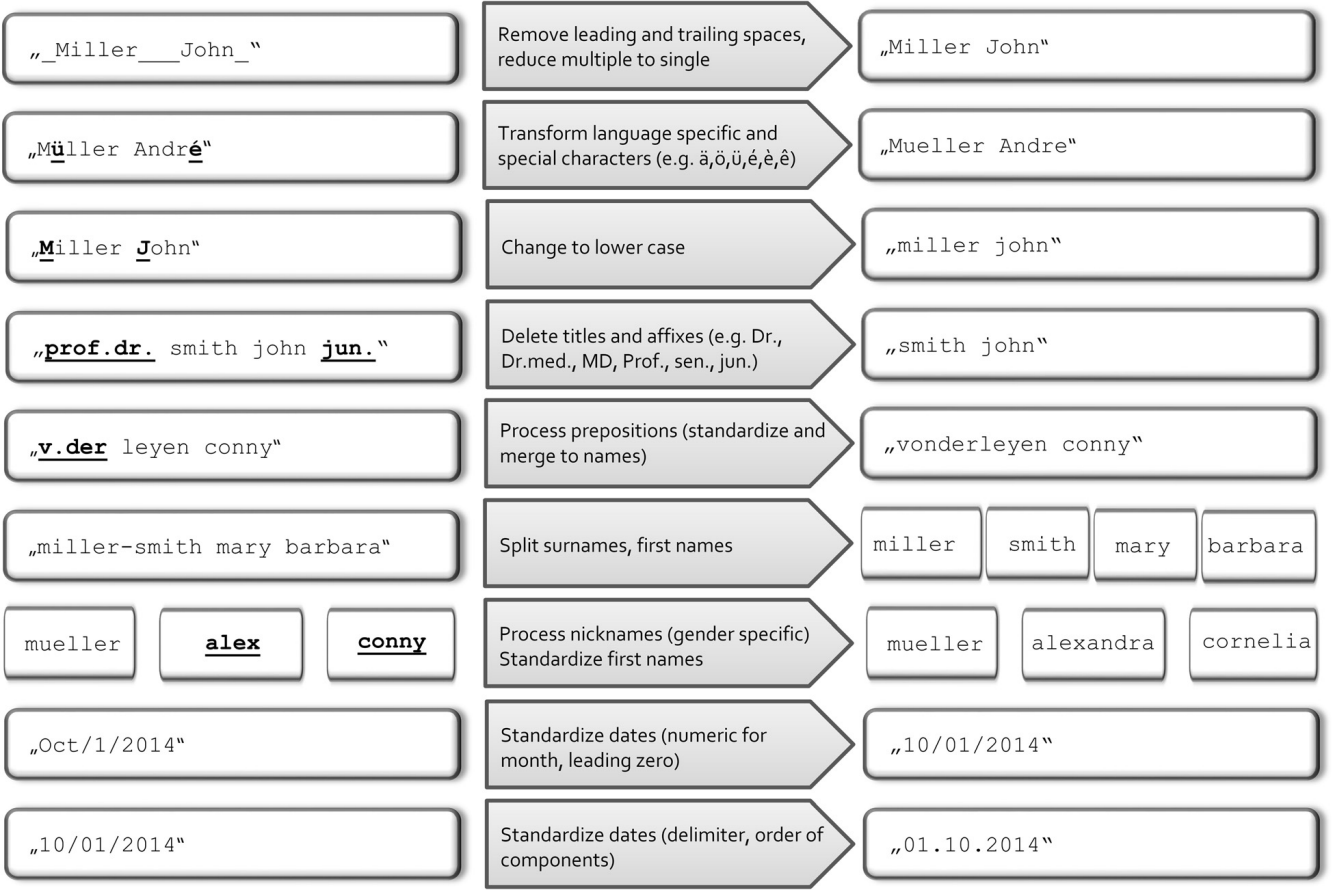
Example of Privacy Preserving Probabilistic Record Linkage (P3RL) pre-processing data cleaning rules

The aim of using templates for pre-processing is to guarantee similar quality and format of linkage variables after data cleaning at each individual site. This is achieved by controlling the data cleaning process with customized pre-processing templates. Templates are created at site C using the information retrieved from the masked data without seeing the plain content. For our projects site-specific templates were created using KNIME Desktop, version 2.7.4 [31, 32]. KNIME is an open source data analytics platform for data mining but any suitable platform or programming language can be used. The pre-processing template consists of a series of data cleaning rules mainly performed using Java regular expressions [33, 34]. Selected basic rules of a pre-processing template and an example of pre-processing names and dates are shown in Fig. 4. The rules standardize names (e.g. transform language specific and special characters), manage pre- and postfixes, split multiple names in separate variables, look up nicknames in gender specific tables and assign uniform missing value characters. Pre-processed dates include checks for the order of date components (e.g. day-month-year vs. month-day-year), numbers instead of names for month, types of delimiters, and leading zero for numeric month and day. Although not particular to P3RL, checking the expected order of numeric day and month is not possible if both are <13.

#### Data cleaning

Data cleaning is required because data from independent sources may differ in many aspects. For example, the format of variables may differ or string variables such as names can be inconsistent due to typographical errors, use of nicknames or abbreviations, changes due to marriage or pre- and postfixes. Therefore, the application of consistent data cleaning rules is crucial for any data warehouse generally [25] and for record linkage particularly [35, 36]. In our P3RL workflow data cleaning is based on pre-processing templates and takes place at sites A&B, before encryption. This step is critical as non-pre-processed linkage variables result in a decreased linkage proportion because true matches are more frequently missed [22].

### Encryption

The aim of encryption is to protect participant privacy and data confidentiality. Encryption is done at the individual sites using an automated encryption tool developed in-house specifically for our P3RL projects. All linkage variables deemed to be confidential (e.g. names, DOB) are encrypted while all other non-sensitive PII (e.g. marital status) are not. In this paper we focus solely on encryption of name and date variables. Nevertheless, the basic method of P3RL is applicable to other variable types. Levels of security and variables to be encrypted will differ from project to project.

#### Encrypting string variables

For P3RL, standard cryptographic procedures such as Keyed-Hash Message Authentication Code (HMAC) [37] cannot be used to encrypt string variables. This is because even tiny differences in string variables produce very different HMAC strings that do not match when compared. For instance, in the case of surnames, the HMAC for “Grün” 301C365327CA3DB972F53EB4CEBC4097F2100A72 is completely different from the HMAC for “Gruen” 5DAB214A7B66680043B6623FA4BE320157EB0E3D. Although “ue” is a common replacement for “ü”, these two surnames would not be identified as potential links by matching HMAC strings. Importantly, name mismatches in health-related data are a common problem [38, 39]. Therefore, the ability to determine the similarity of encrypted name strings instead of using exact HMAC match is critical to the accuracy of P3RL.

**Fig. 5:**
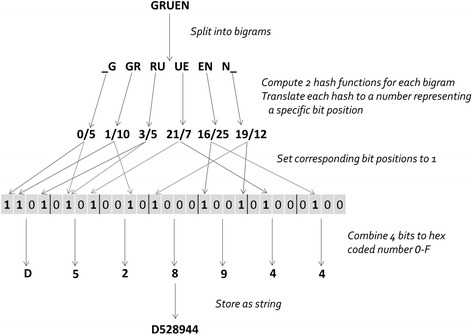
Example of Bloom filter encryption for surname (bigrams, two hash-functions, Bloom filter length 28 bits)

To address this problem we encrypt sensitive PII strings, such as surname, using Bloom filters [14–19]. The use of Bloom filters allows calculation of the similarity of hash codes. Figure 5 shows a detailed example of surname encryption using field-level Bloom filters. In brief, Bloom filters are bit arrays of variable pre-determined length (e.g. 1,000 bits) with all bits initially set to 0. The string (e.g. surname) to be encrypted is split into multiple sets of consecutive letters (q-grams). Based on a secret encryption key a predefined number of hash functions are applied on each q-gram. Each resulting hash is then translated to a number representing a specific bit position on the array where the corresponding bit is changed to 1 [17].

#### Encrypting dates

For P3RL dates are encrypted using simple HMAC’s. Because dates are primarily prone to systematic errors (e.g. swapped day and month, ±1 day differences, transposed numbers like 13 versus 31) they are a common problem for data linkage. A study with SSN comparing DOB from different sources found date errors in 6-25 % of the records [40]. In our work we have found that 17-19 % of all errors in linked DOB are due to ±1 day differences. To address this, we developed a HMAC encryption procedure to handle the most common date errors. It creates two variables, one with plain year and the second with encrypted day, month, full date and full date ±1 day in different sections.

#### Automated encryption tool

The P3RL encryption tool enables data managers at site A and B to encrypt the confidential PII semi-automatically with Bloom filters or HMAC’s. Before starting the encryption, data custodians at site A and B agree on a secret encryption key and a secret test word. The secret key used to generate project-specific hash functions is entered manually at site A and B. First, the secret test word is encrypted using the secret encryption key and is stored as the encryption validation file. Then the confidential linkage variables are encrypted. Encrypted string variables like names are stored as hex coded strings. Four bits of the Bloom filter are translated into one character representing a hex coded number 0 to F to reduce the amount of data (Fig. 5). Finally, the encrypted and unencrypted linkage variables together with the encryption validation file are sent to the linkage center on a secured portable data medium without disclosure of the encryption key.

### Probabilistic record linkage

The last step in the P3RL method is probabilistic record linkage. However, before linkage begins site C must verify that the encryption was performed uniformly at site A and B. If the validation files from site A and B do not match the encryption must be redone before linkage can begin.

We performed probabilistic record linkage using the Generalized Record Linkage System (GRLS), developed by Statistics Canada [41]. We enhanced GRLS by adding additional macros to handle Bloom filters. Similarity of the PII Bloom filter encrypted strings was calculated within GRLS as a Dice coefficient [17, 36] with a range from zero (no similarity) to one (full match). Before calculation of the Dice coefficient, the hex coded strings (Fig. 5) are re-converted into bit arrays for bitwise comparison. GRLS compared all potential site A and B pairs categorizing the probability of a match based on threshold weights as full agreement, partial agreement or disagreement.

Comparison of encrypted string variables was done in three steps. First Bloom filters were compared for full agreement. If not matched then Dice coefficients with different cut-offs representing different levels of similarity (i.e. partial agreement or disagreement) were compared. Lastly, string variables were checked for name transpositions (e.g. swapped first and surname). Dates were also compared in several steps for common errors: (1) plain text year with encrypted day and month for full agreement, (2) encrypted day and month for swapped day and month, (3) encrypted full date and encrypted ±1 day date for ±1 day differences and (4) 1 digit error and transposed numbers in plain year. Additional variables, beyond what is described herein (e.g. addresses), can easily be included in P3RL. Once completed, the link table and the linkage report (i.e. optional information about the linkage quality) is sent to site A and B. Site C deletes all data after the linkage is concluded.

### Ethics

In this paper we describe the P3RL method. No patients were involved and no real data were used, therefore, no ethical approval was needed.

## Discussion

P3RL is a collection of techniques to perform probabilistic record linkage using encrypted sensitive PII data without breaching the confidentiality of the research participants. This paper describes in detail the P3RL method that we have successfully employed for our health-related linkage projects. The implementation of P3RL has allowed reuse of existing health-related data to efficiently answer new and innovative epidemiologic research questions. Furthermore, our P3RL method includes original pre-processing techniques (i.e. masking) that improve the accuracy of linkage procedures. Notably, P3RL is easily customizable and thus applicable in a wide variety of other settings facing similar challenges with the goal of reusing existing data to answer novel research questions.

We faced two main challenges in developing a usable P3RL method. First, we needed to strike a balance between the highly sophisticated methods used in information technology (IT) research and the resources and expertise commonly available in epidemiologic research settings. Second, it was imperative to include techniques that maintained the privacy of PII data throughout the entire P3RL process. The P3RL method described herein overcomes both of these major challenges resulting in a novel and practical solution for linking existing data while preserving participant confidentiality.

Privacy protection using encryption has been extensively researched in the computer science and IT literature. Recently, there have been several studies specifically investigating potential string comparisons using privacy protection measures for record linkage projects [9, 11–13, 42–46]. The methods investigated differ by the quality of the links achieved, the computational requirements and the level of security of the encryption [13, 44]. These methods are generally classified into two-party and three-party protocols [44, 47, 48]. In two-party protocols, two sites apply complex encryption techniques to ensure that no sensitive PII is revealed during the linkage procedures. Two-party protocols are highly secure, as there is no possibility of collusion between one of the sites and the third party. However, they generally are more computationally intensive, therefore, not always feasible for very large datasets [18]. Furthermore, two-party protocols require specific encryption and linkage expertise at the participating sites that may not be available in epidemiologic research settings. Three-party protocols, as described in this paper for P3RL, require a third trusted partner to function as the independent linkage center [20, 36, 42]. The expertise, computing power and control of the linkage process is the primary responsibility of the linkage center. In best practice the linkage center is trained in privacy and security issues and has specialized dedicated resources not likely to be found in traditional epidemiologic research settings. In P3RL the linkage center provides simple tools for the sites for pre-processing and encryption and implements all the probabilistic record linkage procedure. As a result, three-party protocols may be more practicable and easily accepted in epidemiologic research settings.

There are several strengths associated with using our P3RL method for epidemiologic research. We chose Bloom filters for encrypting PII string variables and Dice coefficients to calculate the similarity of the encrypted strings. Research confirms that Bloom Filters [14] are a feasible and efficient method for P3RL, that results in a high quality linkage with reasonable security [13, 17–19]. Bloom filter research has shown similar results to linkage with non-encrypted string variables and superiority to linkage with phonetic encodings [17]. Another strength of P3RL is that the method was developed for health-related research settings, where local personnel often has limited resources, scarce experience in data cleaning and almost never experience in record linkage procedures. We use semi-automated tools, which are easily applied at individual sites and still maintain the participants’ privacy and yield high record linkage quality. To our best knowledge, the masking pre-processing step, which delivers information about the structure of data without revealing the plain content, has not been used in previous research. This step is pivotal for P3RL projects because it allows use of site-specific templates, which facilitate high quality data cleaning at the individual sites without disclosing plain data to the record linkage center. Site-specific templates are crucial, as pre-processing of variables is necessary for high linkage quality, yet data collection at independent sites is rarely standardized [25, 35, 36, 49]. Importantly, which pre-processing procedures to apply and how much pre-processing improves linkage quality before it leads to false positive links is under debate [50, 51]. Many factors such as language specific characteristics (e.g. special characters, common pre- or postfixes) call for individual pre-processing procedures and hamper the development of a gold standard for pre-processing. Another novel step is the validation before the record linkage: As the record linkage center cannot supervise the encryption process at the sites A&B, we included a validation file, which allows to check if both sites applied the same secret key and therefore made the encrypted data comparable, before site C starts the linkage procedures.

Our P3RL method has some limitations. Protecting patient's confidentiality using encryption methods has its price. Masking, pre-processing and encrypting at individual sites and the P3RL linkage process itself are far more time and (human) resource consuming compared to a plain record linkage performed at the record linkage center. Since linkage of encrypted names using Bloom filters is computationally intensive, blocking or filtering techniques to reduce the amount of potential pairs (thus the number of required comparisons) is crucial. Ideally the number of potential pairs should be limited to about 100 million for desktop systems. But caution is needed in trying to estimate computational requirements (Table 1) because estimates depend on the systems and software used for P3RL applications. P3RL projects assume that the sites can manage additional workload, as this method transfers part of the procedures from the linkage center to the sites. Another limitation is that encrypted linkage as in P3RL restricts date comparisons to the most common errors and cannot implement as many date comparisons possible in standard probabilistic linkage projects. For example, swapped digits and single wrong digits in day or month cannot be found without weakening the security of the encryption substantially. The security of any encryption method is always an issue. P3RL method is not designed with any fixed security level. It depends on negotiations between sites and the encryption methods used. Our P3RL method using Bloom filters was developed for research purposes and has not been evaluated for organizational governance requirements. However, Bloom filters have been described and evaluated repeatedly elsewhere. They have been scrutinized and attacked [52, 53] using cryptanalysis and a constraint satisfaction problem (CSP) attack. Even though a frequency attack of Bloom filter encryptions seems not to be completely impossible, the study relies on many assumptions a potential adversary would not find in real world situations, as the authors declare themselves [52]. Furthermore, the assumptions in epidemiological research are different from those in IT research. The privacy of the participants is regulated by ethic review boards designed to protect disclosure to non-approved personnel - not to withstand a CSP attack. Another security issue is the automatic data cleaning and encrypting at sites A and B, where unencrypted data are exported from their main database and stored on notebooks provided by the linkage center with the pre-processing and encrypting tools installed. To overcome this limitation and to ensure that the unencrypted data never leave site A or B, the data custodians can either format the hard drive themselves or destroy the drive used for P3RL. Only a secured mobile device (e.g. USB stick) with the extracted and encrypted data is sent back to the linkage center. Another weakness of privacy preserving linkage methods is that the clerical review of potential but uncertain links is not possible with encrypted variables [49].

**Table 1:** P3RL - Computational requirements of Masking and Shuffling, Pre-processing (100,000 records) and Linkage (100,000 records table A and 50,000 records table B)

Step	Linkage type		
	Plain	P3RL - Encrypted names	P3RL - Encrypted dates
*Mask and shuffle*	-	11 variables to mask and shuffle 5 sec	11 variables to mask and shuffle 5 sec
*Pre-processing* (of variables to be encrypted, source data totally consists of 13 variables)	-	3 name variables to pre-process - > 6 pre-processed name variables built 7 min 54 sec	2 date variables to pre-process - > 2 pre-processed date variables built 35 sec
*Encryption* (source data totally consists of 13 variables)	-	4 name variables to encrypt (trigrams, 10 hash functions, bit array size 800) 1 min 08 sec	2 date variables to encrypt - > 4 date variables built (year plain) 15 sec
*Linkage part 1* -create pairs (filter to reduce potential pairs to 40 Mio)	13 plain variables, 10 rules 1 min 20 sec	9 plain variables, 4 encrypted name variables 1 min 33 sec	13 plain variables 2 encrypted date variables 1 min 23 sec
*Linkage part 2* – apply rules (40 Mio pairs, 10 rules)	63 min	8 Bloom filter array comparisons (two 2x2 matrix rules) 110 min	72 min

Tests were performed on Desktop Computer with Intel® Xeon® CPU, 4 cores, 64-bit, 3 GHz, 12 GB RAM, Windows 7 Professional 64 bit operating system. These estimates were derived using in-house software for masking and encryption, KNIME for pre-processing and G-LINK for linkage. G-LINK is the latest linkage software in desktop version (former GRLS), developed by Statistics Canada

Estimates may vary widely using other programs and/or hardware

## Conclusion

P3RL facilitates the linkage of existing datasets in health-related research settings using automated pre-processing and encrypting to fully protect PII. The privacy of PII is preserved at all times ensuring participant confidentiality is protected. As a result, P3RL expands possibilities for data reuse in settings where regulations restrict access to the PII necessary to link existing health-related datasets. We describe the P3RL method for epidemiological research, however, P3RL is suitable for any other settings with similar challenges. Future studies examining the validity and accuracy of the P3RL method are warranted.

## Abbreviations

CSP: Constraint Satisfaction Problem
DOB: Date of Birth
DOD: Date of Death
GRLS: Generalized Record Linkage System
HMAC: Keyed-Hash Message Authentication Code
ID: Identifier
KNIME: Konstanz Information Miner
P3RL: Privacy Preserving Probabilistic Record Linkage
PII: Personal Identifying Information
SNC: Swiss National Cohort
SSN: Social Security Number
